# Feature Matching Combining Radiometric and Geometric Characteristics of Images, Applied to Oblique- and Nadir-Looking Visible and TIR Sensors of UAV Imagery

**DOI:** 10.3390/s21134587

**Published:** 2021-07-04

**Authors:** Hyoseon Jang, Sangkyun Kim, Suhong Yoo, Soohee Han, Hong-Gyoo Sohn

**Affiliations:** 1 School of Civil and Environmental Engineering, Yonsei University, Seoul 03722, Korea; hyoseon9026@yonsei.ac.kr (H.J.); sankun@yonsei.ac.kr (S.K.); swennoir@yonsei.ac.kr (S.Y.); 2 Department of Geoinformatics Engineering, Kyungil University, Gyeongsan 38428, Korea; scivile2@gmail.com

**Keywords:** thermal infrared (TIR) oblique image, geometry, wavelength, phase congruency, histogram matching, Image Matching by Affine Simulation (IMAS), Unmanned Aerial Vehicle (UAV)

## Abstract

A large amount of information needs to be identified and produced during the process of promoting projects of interest. Thermal infrared (TIR) images are extensively used because they can provide information that cannot be extracted from visible images. In particular, TIR oblique images facilitate the acquisition of information of a building’s facade that is challenging to obtain from a nadir image. When a TIR oblique image and the 3D information acquired from conventional visible nadir imagery are combined, a great synergy for identifying surface information can be created. However, it is an onerous task to match common points in the images. In this study, a robust matching method of image pairs combined with different wavelengths and geometries (i.e., visible nadir-looking vs. TIR oblique, and visible oblique vs. TIR nadir-looking) is proposed. Three main processes of phase congruency, histogram matching, and Image Matching by Affine Simulation (IMAS) were adjusted to accommodate the radiometric and geometric differences of matched image pairs. The method was applied to Unmanned Aerial Vehicle (UAV) images of building and non-building areas. The results were compared with frequently used matching techniques, such as scale-invariant feature transform (SIFT), speeded-up robust features (SURF), synthetic aperture radar–SIFT (SAR–SIFT), and Affine SIFT (ASIFT). The method outperforms other matching methods in root mean square error (RMSE) and matching performance (matched and not matched). The proposed method is believed to be a reliable solution for pinpointing surface information through image matching with different geometries obtained via TIR and visible sensors.

## 1. Introduction

Thermal infrared (TIR) images are acquired in the approximate range of 9 to 14 μm of the electromagnetic spectrum and applied to various fields, such as 3D building modeling and management [1,2], diagnostics related to fire [3] and heat loss [4], disaster management (e.g., an earthquake [5] or volcano [6]), a military field that detects abnormalities [7], and the monitoring of safety facilities (e.g., a nuclear power plant) [8]. In this spectral range, it is possible to obtain information even at night, unlike with visible images. TIR images have been widely adopted because they allow for the continuous monitoring of problems that current cities are facing, which can occur at any time of the day [9,10]. However, TIR images have a much lower resolution compared to visible images. Due to this limitation, it is usually difficult to pinpoint the area we are interested in with TIR imagery alone. To overcome this hurdle, a convergent analysis approach combining both TIR and visible images, including high-accuracy location information, could be essential. During the course of image convergence, image matching between visible and TIR images needs to take place to identify the corresponding points of interest (POIs).

Scale-invariant feature transform (SIFT) [11] and speeded-up robust features (SURF) [12] are the most representative image matching methods. SIFT first constructs a Gaussian scale space and extracts feature points, interpreting features using the gradient histogram technique. SURF derives feature points based on the Hessian matrix and introduces an integrated graph technique to enhance efficiency. Verykokou and Ioannidis [13] utilized the SURF detector to perform matching on oblique images acquired with a visible sensor. Jiang and Jiang [14] applied SIFT detector to execute matching for visible sensor-based oblique images. SIFT and SURF were initially proposed to find matching points in visible images, but they have also been applied to find matching pairs in visible and TIR images.

Ricaurte et al. [15] studied the performance of feature point detection and description between long-wave infrared and a visible dataset obtained from a cross-spectral stereo rig. The resolutions of the visible and long-wave infrared images were 658 × 492 and 640 × 480, respectively. They evaluated the performance of algorithms under two major domains: based on image derivatives (SIFT and SURF), and based on image intensities (Oriented FAST and Rotated BRIEF (ORB) [16], Binary Robust Invariant Scalable Keypoints (BRISK) [17], Binary Robust Independent Elementary Features (BRIEF) [18], and Fast Retina Keypoint (FREAK) [19]). They concluded that SIFT performs the best in most evaluation categories, such as rotation, scale, blur, and noise.

Aguilera et al. [20] considered the feature point descriptor rather than detection and matching as the key element when finding correspondences from visible and long-wave infrared spectrum images with SIFT and its modification. They proposed the use of an edge-oriented histogram (EOH) descriptor considering the non-linear relationship between pixel intensities. The results showed better matching accuracy compared to SIFT and SURF alone and realized the importance of using histograms of contour orientations in the neighborhood of the given key points. All of these studies attempted to match visible and TIR images in the spatial domain.

Recently, applying phase congruency (PC) based on a frequency domain for matching visible and TIR images has been studied. Mouats et al. [21] adopted PC as a feature detector and generated edge maps of visible and TIR images. Descriptors are computed based on the EOH descriptor and combined with the Log-Gabor coefficients calculated in the previous step. This involved setting up a multispectral stereo rig composed of a visible and TIR sensor mounted on a car’s roof and capturing multi-modal image pairs. The resolutions of the visible and TIR images were 658 × 492 and 640 × 480, respectively. The feature correspondence results in their research indicated that intensity-based algorithms (SIFT, SURF, ORB, and BRISK) provided poor correspondence in the multispectral scenario.

Liu et al. [22] utilized PC as a feature detector for visible image and long-wave infrared image matching. They applied the maximum and minimum moments of PC to the original image and Gaussian-smoothed images for corner detection, respectively, and then combined two images to create enhanced moments of PC. They extracted overlapping subregions using Log-Gabor filters to generate descriptors. The image size they used was 639 × 431 pixels for both visible and long-wave infrared images. The experimental results show that the accuracy rate is 50% higher than those of traditional approaches, such as the EOH descriptor, the phase congruency edge-oriented histogram descriptor (PCEHD), and the Log-Gabor histogram descriptor (LGHD) algorithms.

These efforts have been conducted in the spatial and frequency domain for matching visible and TIR images. However, the studies mentioned above were obtained from the same geometry and focused on the city’s ground image, including many objects. Methods for image matching with different geometries based on visible sensors have since been designed, such as principal component analysis–SIFT (PCA–SIFT) [23], affine SIFT (ASIFT) [24], iterative SIFT (ISIFT) and iterative SURF (ISURF) [25], MSER–SIFT (MMSIFT) [26], Matching On Demand with view Synthesis (MODS) [27], and the mixture-feature Gaussian mixture model (MGMM) [28]. Amanda et al. [29] utilized an ASIFT detector to match images with different geometries based on a visible sensor.

Most recently, Image Matching by Affine Simulation (IMAS) was developed by Rodríguez et al. [30] as a method of developing ASIFT, and it was utilized to match different geometry images obtained from an Unmanned Aerial Vehicle (UAV) by Jang et al. [31]. The pivotal contents of IMAS are primarily composed of three characteristics. First, it is the near-optimal α°-covering of the feature detector. The α°-covering is based on the transition tilt theory and creates an image through simulation to consider images of assorted angles. At this time, stereographic projection, which is a map projection based on a quaternion angle, is applied. The second major characteristic of IMAS is the creation of hyper-descriptors in the feature descriptor. The hyper-descriptor produces a cluster based on a myriad of feature points extracted from images of various angles through the near-optimal α°-covering and then creates a descriptor for the cluster. These hyper-descriptors can improve the operation speed of the image matching process. The last part of the IMAS is a contrario model of the feature descriptor process. This model is a parameter tuning method and is applied to increase matching pairs. In conclusion, IMAS has the potency of robust extracted feature points for different geometry images.

In the intensive literature review, it was difficult to find any study that attempted to match images between different geometries and different spectral characteristics, for example, visible nadir-looking vs. TIR oblique and visible oblique vs. TIR nadir-looking imagery. We determined that, compared to the rapid increase in the effectiveness of TIR images, there are relatively few studies that attempt to fuse them with visible images. Additionally, there are no appropriate datasets available for matching. Accordingly, we carefully designed for data acquisition processes to fit our objectives using UAV imagery of building and non-building areas. A new image matching method is proposed for oblique and nadir-looking images acquired through the UAV’s visible and TIR sensors. In this work, we propose our phase congruency with the histogram–IMAS (PCH–IMAS) method.

The remainder of this study is organized as follows. Section 2 describes the image matching method proposed in this study, and Section 3 illustrates the optimal selection of experimental location and data acquisition processes through UAVs for maximizing research purposes. Section 4 shows the matching experimental results (including related interpretations), and Section 5 expresses the conclusion.

## 2. Methodology

**Figure 1:**
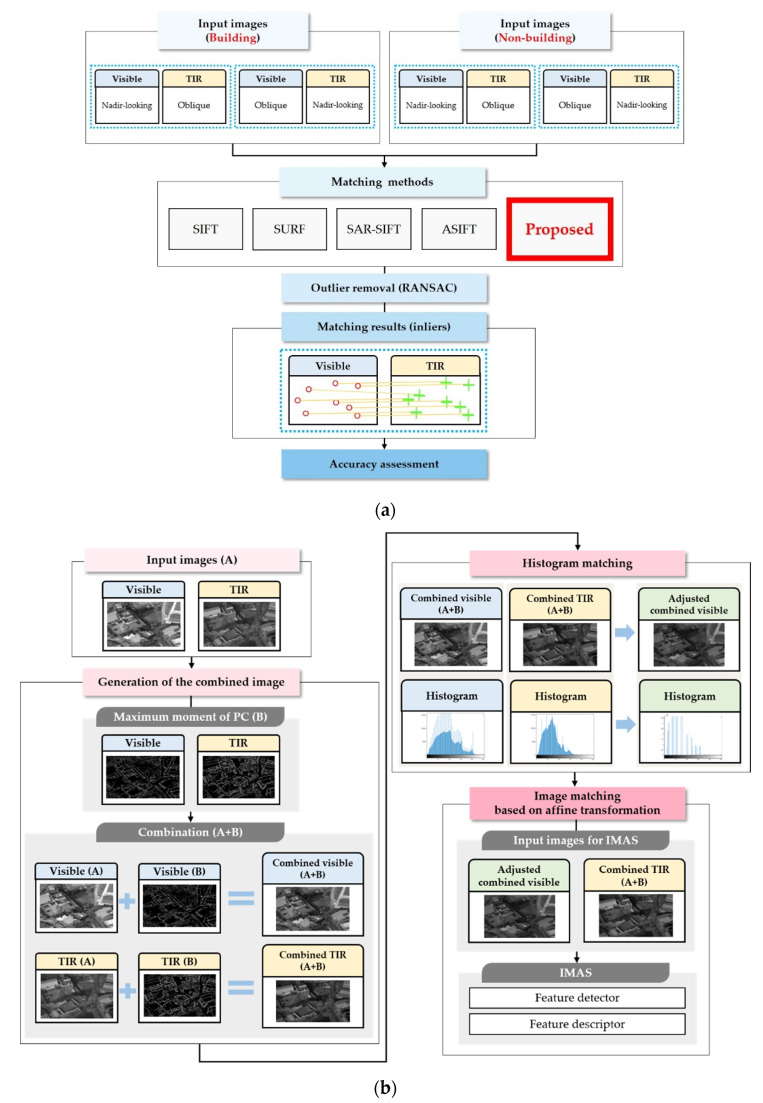
Research flow: (**a**) macroscopic frame of method; (**b**) detailed flow chart of the proposed (red square box in (**a**)) method in this study.

Figure 1 is a flowchart showing the research approach. The major steps, from inputting the test images to evaluating the inliers’ accuracy, are shown in Figure 1a. Visible nadir-looking vs. TIR oblique and visible oblique vs. TIR nadir-looking image sets were the inputs for building and non-building data types, respectively. A total of 5 matching methods were applied for these 4 image sets, including the method proposed in this study. Afterward, the inlier was finally obtained through outlier removal, and an inlier accuracy assessment was performed.

Figure 1b is a detailed description of the matching method proposed in this study. It represents the ‘proposed’ part (highlighted in the red square box) of Figure 1a. Only the visible images corresponding to the TIR image region were selected and used in the subsequent experiments. Moreover, after converting the RGB of the visible image to grayscale, a proposed matching method was conducted.

Our principal concept consists of three parts. First, the combined images are generated from visible and TIR images. A combined image means it is an edge-enhanced image that has been created by incorporating edges extracted from the original visible and TIR imagery. The edges in the combined images are created with the maximum moment of the PC in the frequency domain, considering the non-linear relation of pixel intensities between the visible and TIR images in the spatial domain. Second, the histogram of the combined visible image is adjusted based on the histogram of the combined TIR image, considering the pixel values in the TIR images that contain invariant characteristics relative to the sun’s illumination of objects. Third, IMAS is joined to improve the geometric barrier between the nadir and obliqueness of visible and TIR imagery. A detailed explanation of each step is presented in the subsequent sections.

### 2.1. Generation of the Combined Image Based on Edge Information in the Frequency Domain

The combined images are devised to solve the non-linear relationship between pixel intensities for visible and TIR images. The combined image is an edge-enhanced image that has been created by combining edges extracted from the original image with the maximum moment of the PC in the frequency domain into the original image in the spatial domain. The pixel values of the extracted edges are 255, which converts the corresponding pixel values of the original images through the combined process. This process can reduce the probability that two images with different wavelengths will be recognized differently for the same object.

PC is a feature extraction method using only phase information in the frequency domain. Oppenheim and Lim [32] proposed the basic concept of PC. They claimed that phase information is more crucial than amplitude information where image analysis is concerned. Morrone and Owens [33] proposed mathematical procedures of PC through Fourier series expansion at the signal location. The Log-Gabor filter is currently embraced by Kovesi [34] to extract the image features, being robust to changes in the image’s orientation and scale. Kovesi [35] finally completed the formula for PC, as shown in Equation (1).

(1) $$PC_{n}\left(x,y\right)=\frac{\sum_{n}W\left(x,y\right)\lfloor A_{n}\left(x,y\right)\left[\cos\left(\phi_{n}\left(x,y\right)-\bar{\phi }\left(x,y\right)\right)-\left|\sin\left(\phi_{n}\left(x,y\right)-\bar{\phi }\left(x,y\right)\right)\right|\right]-T\rfloor ;}{\sum_{n}A_{n}\left(x,y\right)+\varepsilon }$$

where W(x,y) is a weighting factor for the frequency spread, $A_{n}$ represents the amplitude of the *n*-th Fourier component, and $\phi_{n}\left(x,y\right)$ is the local phase of the Fourier component at the location. The value of $\bar{\phi }\left(x,y\right)$, maximizing this equation, is the amplitude weighted mean local phase angle of all Fourier coefficients at the considered point. T is counted as a noise threshold from the statistics of the Log-Gabor filter in the image. Only values exceeding the calculated T can be finally meaningful values. Furthermore, a small constant ε is used to avoid division by zero. ε is set to 0.0001 in PC.

In this study, the maximum moment of PC was elicited by the covariance matrix (Equations (2) and (3)). This is calculated to produce a highly localized operator, which is used to identify edges in invariant positions compared with surrounding pixels.

(2) Covx(θ)=PC(θ)cos(θ)

(3) Covy(θ)=PC(θ)sin(θ)

where PC(θ) is the phase congruency value determined at the orientation, θ. In this study, the maximum moment of PC was computed through Equations (4)–(7).

(4) $$\mathrm{Maximum}\;\mathrm{moment}\;\mathrm{of}\;PC=\frac{1}{2}\left(a+c+\sqrt{b^{2}+{\left(a-c\right)}^{2}}\right)$$

(5) $$a=\sum^{\;}Covx{\left(\theta \right)}^{2}$$

(6) $$b=2\sum^{\;}Covx\left(\theta \right)Covy\left(\theta \right)$$

(7) $$c=\sum^{\;}Covy{\left(\theta \right)}^{2}$$

**Figure 2:**
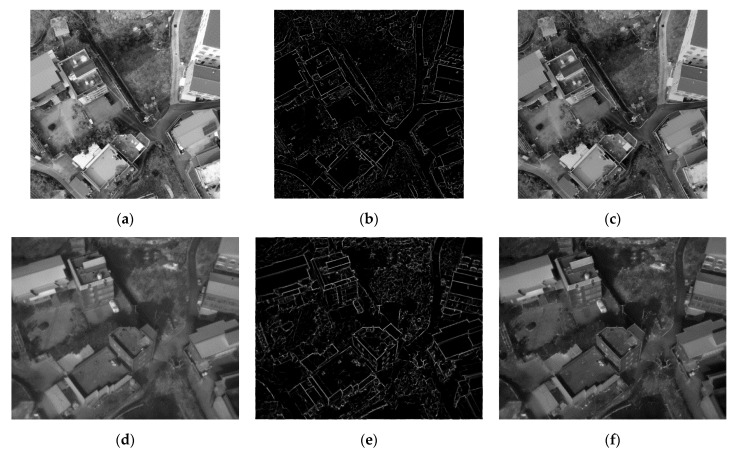
Generation of the combined image: (**a**) original visible image; (**b**) maximum moment of PC extracted from the visible image; (**c**) result of the combination of (**a**,**b**); (**d**) original TIR image; (**e**) maximum moment of PC extracted from the TIR image; (**f**) result of the combination of (**d**,**e**).

The maximum moment of PC obtained through the above processes implies edges in the image. Furthermore, from a preceding test, a good matching result could not be expected solely by the maximum moment of PC without considering the features based on the pixel values of the original image. Thus, in this study, the maximum moment of PC was combined with the original image. We elected to use the method concerning all pixel values in the original image and the PC’s features. Figure 2 illustrates the process of generating a combined image. Figure 2a shows the original visible image, Figure 2b indicates the maximum moment of PC extracted from the original image, and Figure 2c displays the combination of both. Figure 2d–f shows the same results for TIR images.

Finally, the combined image that was created through the maximum moment of PC contains the information of similar features, even with different wavelengths. Thus, they are accepted as the same instruction in the image recognition process. The combined image is an acceptable solution for the limitation of matching between the visible and TIR images.

### 2.2. Histogram Matching

Histogram matching is used to consider the pixel values of TIR images that include invariant characteristics relative to the sun’s illumination of objects. The advantages and disadvantages of TIR and visible sensors are complementary. For example, the TIR sensor can get information in a nocturnal environment, but a visible sensor can provide much better information in a well-lit environment. This is due to the fact that the passive TIR sensor is entirely reliant on the object’s thermal radiation [36]. Additionally, TIR images contain texture information, which is essential for distinguishing objects and recognizing surroundings [36]. We consider pixel values in TIR images as absolute values representing the unique physical properties of the objects in images. In this study, histogram matching adjusted the combined visible image, similar to the combined TIR image histogram distribution. Therefore, performing the match with the adjusted combined visible image and the combined TIR image increases the probability of matching through the derivation of robust feature points that are not affected by wavelength changes.

**Figure 3:**
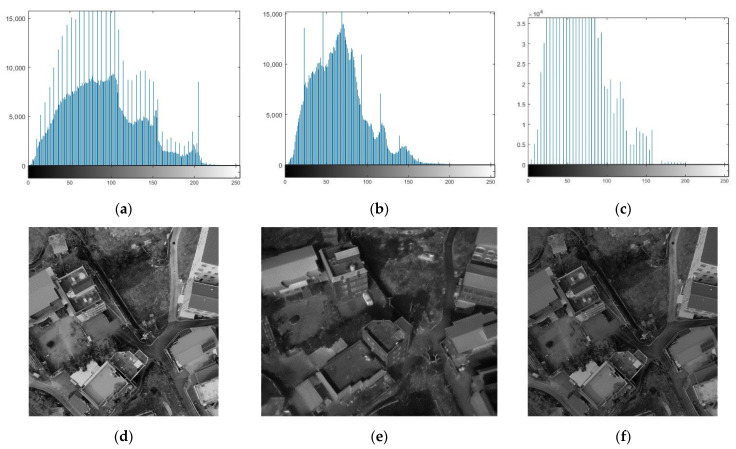
Histogram matching: (**a**) histogram of combined visible image; (**b**) histogram of combined TIR image; (**c**) adjusted histogram of the combined visible image; (**d**) combined visible image; (**e**) combined TIR image; (**f**) adjusted combined visible image.

Histogram matching, also called a color transfer, is widely employed in image processing [37], such as image contrast control and stitching [38,39,40]. In this study, histogram matching was applied to modify the contrast or brightness of the images with wavelength differences [41,42]. Figure 3a–c shows examples of the histogram of the combined visible image in Figure 3d, the combined TIR image in Figure 3e, and the adjusted combined visible image in Figure 3f, respectively.

Eventually, the combined visible image’s histogram pattern was regulated similarly to the combined TIR image. The radiometric difference between the visible and TIR images diminished significantly through the series of processes described above, and only the geometric disparity in the images remained.

### 2.3. Image Matching Technique Based on Affine Transformation

In the final part of our method, IMAS was used to improve the matching limitation between visible and TIR images with different geometries. This matching method is based on affine transformation and was proposed by Rodríguez et al. [30]. The affine transformation includes both linear and similarity transformations. In other words, it preserves isotropic scaling and parallelism. Additionally, IMAS can carry shear and reflection as well as rotation, translation, and scaling. IMAS has a high potential for sturdy matching for different geometry images. The crucial part of IMAS is the near-optimal α°-coverings, which aim to shape an image analogous to that acquired from diversified angles. For this task, α°-coverings are similar to lines of latitude and longitude in stereographic projections.

**Figure 4:**
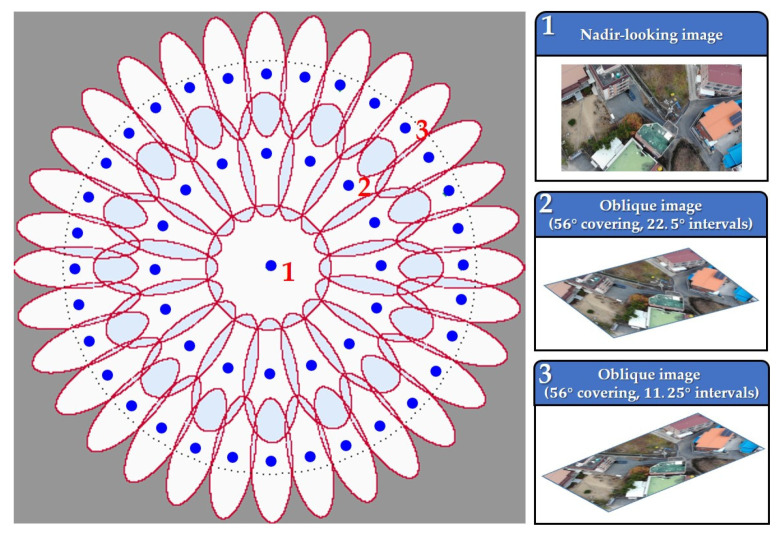
The α°-covering in polar coordinates and images obtained from the blue dots of disks 1, 2, and 3.

The α°-covering is expressed as shown in Equation (8) and Figure 4.

(8) α°-covering=∪B(S,r)

where Β is a disk, which indicates the white circles and ellipses separated by red borders in Figure 4. S is the center of the covered area, which is the position of the blue dots in Figure 4. r is the radius of the disk. Thus, α°-covering is the union of disks created based on each blue dot.

Furthermore, the case of S can be shown in more detail, as shown in Equation (9).

(9) $$S=\left\{[T_{t}R_{\emptyset }|t\leq \frac{1}{cos\left(\gamma {}^{\circ}\right)}\right\}$$

where $T_{t}$ and $R_{\emptyset }$ are calculated by the transition tilt theory. $T_{t}$ is the latitude that determines the levels for the locations of disks 1, 2, and 3 marked in Figure 4. $R_{\emptyset }$ is the longitude that indicates a change in the position of a disk with the same latitude. Lastly, $\gamma^{{}^{\circ}}$ is the angle. If t=1, arccos (1)=0, which indicates the image viewed from nadir by disk 1. On the other hand, r of Equation (8) is expressed in detail, as shown in Equation (10).

(10) $$r=\left(log\frac{1}{cos\left(\alpha {}^{\circ}\right)}\right)$$

where α° is the angle, which calculates the size of the disk and determines the area where a single image can be covered. Finally, the α°-covering is generated, as shown in Figure 4, through the process mentioned above. In this study, we applied a 56°-covering. As a result, we have created nadir-looking images of disk 1 and oblique images of disks 2 and 3 with the α°-covering in polar coordinates, as shown in Figure 4. At this time, we input the actual image acquired in this study in disk 1, and disks 2 and 3 are the images calculated through the α°-covering.

The α°-covering can simulate images acquired from various angles by changing the geometry of the camera. In Figure 4, the blue dots indicate the positions according to the latitude and longitude of the camera. For example, if the image is acquired from disk 1, located in the covering center, we can obtain the nadir-looking image. Disks 2 and 3, located around disk 1, produce oblique images. Therefore, coverings were made at 22.5° intervals in disk 2, and a total of 16 oblique images were created. In addition, coverings were made at 11.25° intervals in disk 3, and a total of 32 oblique images were produced in this study.

### 2.4. Outlier Removal

The random sample consensus (RANSAC), proposed by Fischler and Bolles [43], was implemented to remove outliers included in the matching result. The goal of this step can be achieved by repeating the following two steps. First, a sub-dataset is randomly selected from the original dataset. Then, the model and model parameters for the picked sub-dataset are determined. Second, the system verifies how well the previously computed model parameters correlate with all the data. If the data do not fit the given model, they are segregated as an outlier. Additionally, if they match the given model, they are considered as an inlier. The set of valid data attained from the fitting model is labeled a consensus set. The RANSAC algorithm reiterates the two steps above until enough consensus sets are obtained.

## 3. Optimal Selection of Experimental Environment

The first criterion considered in this study for selecting logical locations is to include both urban and rural characteristics. In addition, the location to be selected must be within the UAV operating permit radius. When we acquired images through visible and TIR sensors, we needed an area where various objects with different shapes expressed according to wavelengths were mixed. It is a better condition if not only in form but also in a place where several subjects with different textures exist together. The angle of the sensors is adjusted to get different geometry images, but it is more reasonable if there are factors around the area that cause changes in topography, such as a mountain.

On the other hand, as mentioned in the introduction, there were no appropriate datasets available for our research purpose. Therefore, we carefully designed the data acquisition processes. The primary considerations were the universality of UAV operation, weather conditions, the angle of the sensor for oblique image acquisition, and flight altitude. Finally, we selected the optimal location and acquired suitable datasets. These processes are described in detail in the subsection below.

### 3.1. Considerations for an Optimal Research Location

**Figure 5:**
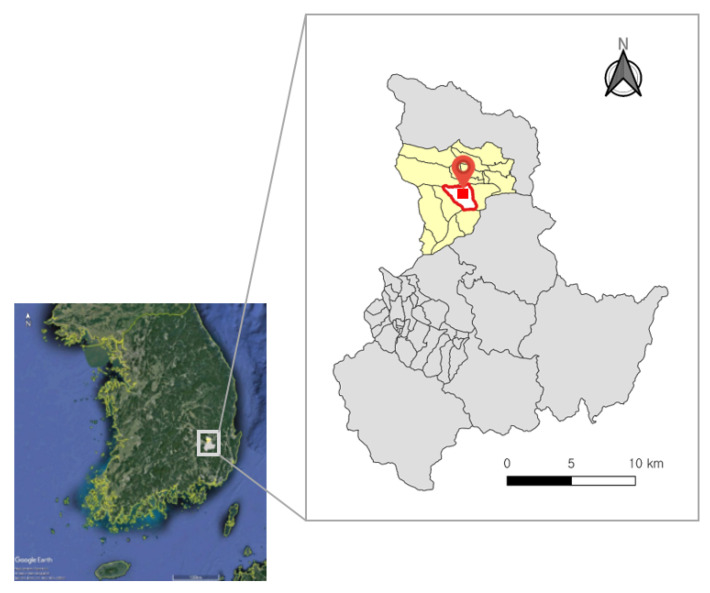
Location of the study area (red boundary) and data acquisition field (red quadrangle) depicted in Google Maps showing South Korea (inset).

Buho-ri was selected as the optimal environment for this research. Buho-ri is located in Gyeongsan-si, Gyeongsangbuk-do, South Korea, and covers about 3.01 km^2^, composed of townhouses and agricultural fields. The shape of roofs varies greatly and includes squares and polygons. The arrangement of roads and buildings is irregular, as is common in unplanned towns. Buho-ri has a variety of objects, such as furrows, bushes, and twigs, that can express various textures in the images. Additionally, the area features mountains in the northwest, causing changes in topography. In this study, images of a total of about 0.06 km^2^ were acquired in the areas in which the houses and fields are concentrated. The red square in Figure 5 indicates the image acquisition area.

### 3.2. Data Acquisition Processes for Maximizing Research Purpose

The data acquisition that maximizes our research purposes was designed based on the following details. We obtained four kinds of images (visible nadir-looking, visible oblique, TIR nadir-looking, and TIR oblique) on 26 November 2020. On the day of the image acquisition, the temperature reported was from 4.9 to 13.3 °C, and the wind speed was 6.5 km/h. Additionally, there was no rain or snow in the area, but a slight haze occurred. TIR nadir-looking and oblique images were acquired from noon to 2 p.m., and visible nadir-looking and oblique images were gained until 5 p.m., consecutively.

**Figure 6:**
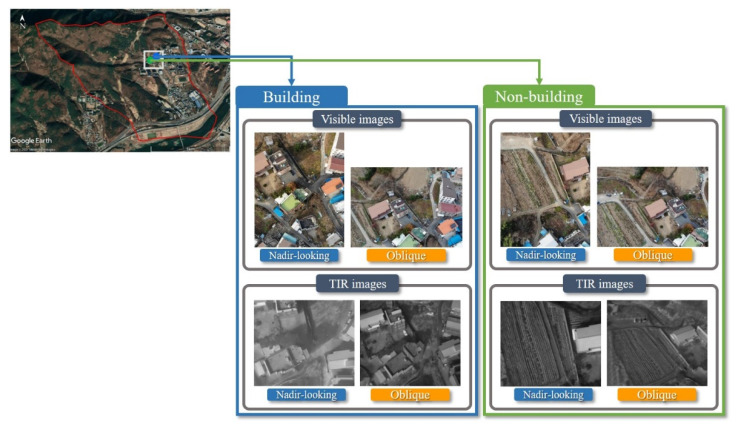
Classification of building and non-building types for obtained images (visible nadir-looking, visible oblique, TIR nadir-looking, and TIR oblique) on Google Maps.

Figure 6 shows the amassed images classified into building and non-building areas for the matching experiments. The red boundary of the Google Maps in Figure 6 is Buho-ri, and the white square is the image acquisition district. Additionally, the blue and green dots are the building and non-building zones, respectively.

#### 3.2.1. Acquisition of Nadir- and Oblique-Looking Visible Images

The acquisition of visible images is divided into two parts (nadir-looking and oblique). Table 1 shows the details related to image acquirement. The nadir-looking images were obtained at an angle of 90° facing the ground. The oblique images were gained by tilting the camera gimbal by 30° from the plumb line. As a result, we obtained a total of 60 nadir-looking images and a total of 85 oblique images of the study area.

#### 3.2.2. Acquisition of Nadir- and Oblique-Looking TIR Images

Table 2 is a detailed description of the acquisition of TIR images. The Zenmuse XT camera’s spectral range is from 7.5 to 13.5 µM, and the temperature ranges from −25 to 135 °C. The TIR images, which observe the object’s temperature properties, were characterized by the pixel values for gas pipes and water pipes made of steel appearing closer to 255 than the surrounding pixels. These properties differ from pixels in the visible image, containing only information about the object’s shape depending on the sun’s illumination. Nadir-looking and oblique images were acquired in the same way as the visible images. We obtained a total of 91 nadir-looking images and 108 oblique images.

## 4. Experimental Results and Discussion

The five matching methods mentioned above were applied for images with different wavelengths and geometries, and the results were compared. Reasonable matching methods (SIFT, SURF, synthetic aperture radar–SIFT (SAR–SIFT), and ASIFT) have been judiciously selected to compare the performance and accuracy of the proposed method. These four methods provide reliable source codes that are necessary for the quantitative comparison of matching results. SIFT and SURF were administered based on OpenCV, to allow for efficient handling. SAR–SIFT was made and is shared by multiple users via GitHub. Moreover, ASIFT can be downloaded by following the hyperlink in the author’s paper, which is highly trustworthy.

In addition, the usability of the matching method is important in comparing the performance and accuracy of the proposed method. SIFT and SURF are the most representative methods of matching between visible and TIR images. We chose SIFT and SURF as the generalized framework of the matching method and applied them. SAR–SIFT was used for image matching with different wavelengths and different geometries [44]. This is the most relevant category that we wanted to investigate in this study. Lastly, ASIFT is a commonly used method for matching different geometries. Recently, it has also been tested for distinct wavelengths [45]. We hypothesized that ASIFT could be used not only for UAV image matching with substantial geometric differences but also for visible and TIR images.

The root mean square error (RMSE), a widely used indicator for evaluating image matching accuracy, was calculated for accuracy assessment [46,47,48,49]. We estimated 2D-affine transform coefficients based on 25 feature points that were chosen by manual selection for every image. It is assumed that the 2D-affine transform coefficient estimated through manual selection is the true value of the transform gained as a result of matching the image. Then, the 2D-affine transform coefficients were counted based on the inlier feature points obtained through the five matching methods applied in this study. Finally, we measured the distance between the transform coefficient based on the true value determined previously and the transform coefficient based on the inlier feature points. The measurement unit is a pixel, and the smaller the measured value, the higher the accuracy.

The verification of the proposed algorithm has been under various environments, such as a city, where many buildings are placed, and rural areas with relatively few buildings and many fields. Therefore, matching experiments were performed for four different image cases, as shown in Table 3. Finally, we selected the most effective matching method through an accuracy assessment of the matching results.

This experiment’s hardware specifications were Intel(R) Core(TM) i5-8500 CPU @ 3.00 GHz, and 32 GB RAM, and they were the same for all methods. The software environments and languages were diverse for each matching method, as shown in Table 4.

### 4.1. Comparison of Matching Results

**Figure 7:**
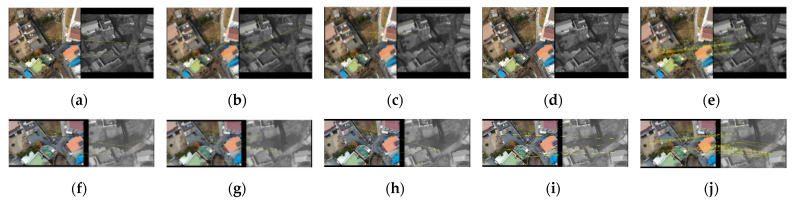
Matching results of building type images: visible nadir-looking (630 × 627) vs. TIR oblique (640 × 512) case: (**a**) SIFT, (**b**) SURF, (**c**) SAR–SIFT, (**d**) ASIFT, and (**e**) proposed method; visible oblique (731×433) vs. TIR nadir-looking (635×436) case: (**f**) SIFT, (**g**) SURF, (**h**) SAR–SIFT, (**i**) ASIFT, and (**j**) the proposed method.

**Figure 8:**
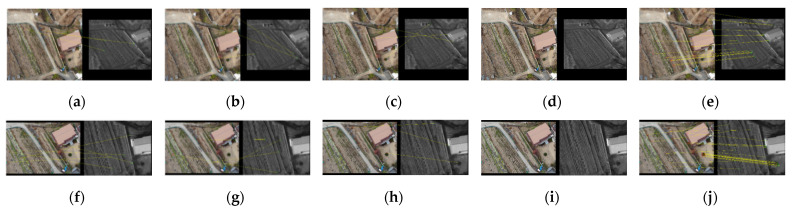
Matching results of non-building type images: visible nadir-looking (659 × 629) vs. TIR oblique (545×437) case: (**a**) SIFT, (**b**) SURF, (**c**) SAR–SIFT, (**d**) ASIFT, and (**e**) proposed method; visible oblique (669×460) vs. TIR nadir-looking (605×494) case: (**f**) SIFT, (**g**) SURF, (**h**) SAR–SIFT, (**i**) ASIFT, and (**j**) the proposed method.

Figure 7 and Figure 8 are arrangements of the results according to the five matching methods (SIFT, SURF, SAR–SIFT, ASIFT, and the proposed method) for building and non-building types. The sizes of the images were recorded together. First, Figure 7a–e and Figure 8a–e show visible nadir-looking and TIR oblique results. SIFT, SURF, SAR–SIFT, and ASIFT did not match regardless of the presence or absence of buildings, as shown in Figure 7a–d and Figure 8a–d. However, as shown in Figure 7e and Figure 8e, the proposed method’s results accomplished excellent matching in both building and non-building types.

On the other hand, Figure 7f–j and Figure 8f–j show the matching results between the visible oblique and the TIR nadir-looking images, which have the opposite geometry compared to the previous one. As presented in Figure 7j and Figure 8j, the proposed method was the only one successful.

Table 5 indicates the number of inliers of the matching results presented in Figure 7 and Figure 8. When SIFT, SURF, SAR–SIFT, and ASIFT were applied, they made no match. Therefore, there was no inlier derived through the four matching techniques. Only the number of inliers following using the matching method proposed in this study was meaningful.

As shown in Table 5, when comparing the building type with the non-building type, the number of inliers of the building type increased by approximately 7–10. Building-type images had many points where differences in pixel values were evident from various objects. Thus, they were inclined to extract a more significant number of feature points. However, non-building images consisted of fields similar to the bare ground environment. Most of these terrains had the same pixel value distribution, and they were less likely to be extracted as a feature point. Therefore, the multiplicity of objects in the two images occasioned a gap between the number of inliers.

### 4.2. Grasping the Characteristics of Extracted Feature Points

The matching method proposed in this study became the only solution for image matching in all cases. We aimed to understand the conditions under which the proposed method extracts robust features. Therefore, we confirmed the location characteristics of feature points in certain circumstances. Through this, when UAV images acquired from various settings are secured, it is possible to select images that can be matched preferentially. Additionally, we can evaluate and choose an image acquisition area and surroundings that can improve matching accuracy based on the characteristics of the feature.

**Figure 9:**
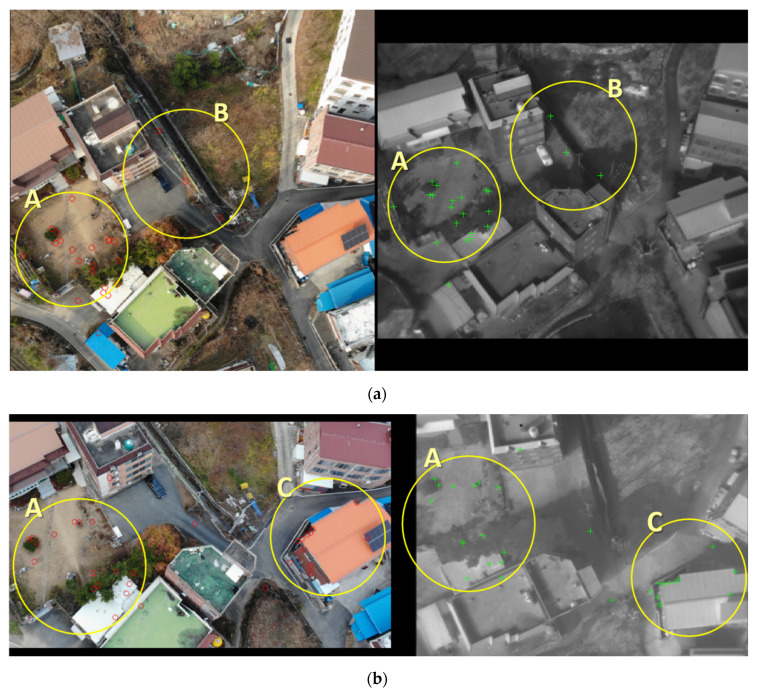
Understanding the characteristics of feature points of the building type: (**a**) visible nadir-looking vs. TIR oblique case; (**b**) visible oblique vs. TIR nadir-looking case.

**Figure 10:**
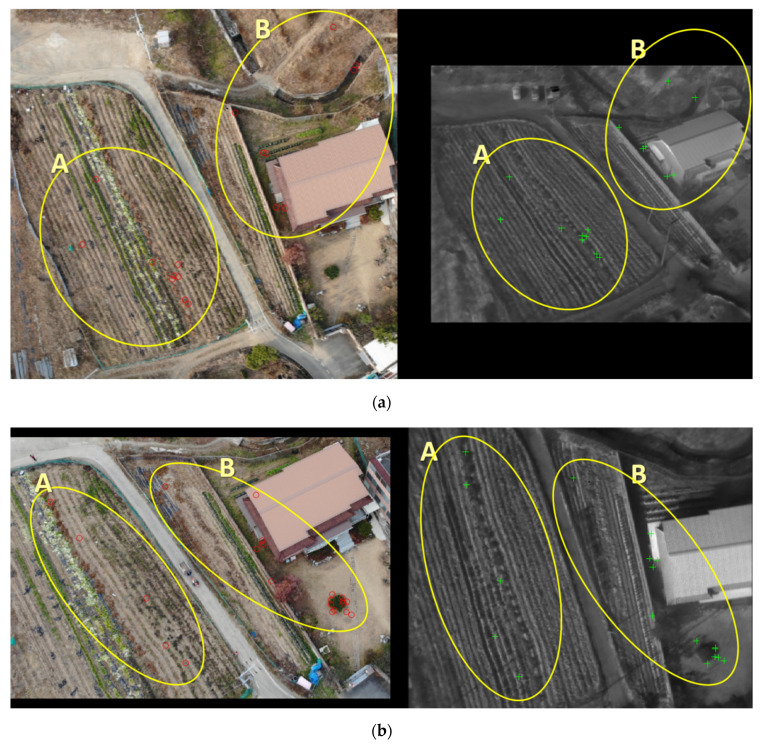
Understanding the characteristics of feature points of the non-building type: (**a**) visible nadir-looking vs. TIR oblique case; (**b**) visible oblique vs. TIR nadir-looking case.

Figure 9 and Figure 10 are feature points of building and non-building types, respectively. First, Figure 9 is a building-type image with diversiform housing. In general, building images in nadir view have many edges or corners; a large amount of these factors can be revealed as feature points. However, the proportion of feature points presented from the oblique image was bigger on the ground than in the building’s edge or corner. We hypothesized that ground features were robust to changes in geometry and wavelengths and less sensitive to affinity. We handled each factor by grouping feature points into areas A, B, and C, according to the level of description and the distribution location of the points.

Figure 9a shows the feature points for the building type acquired from the visible nadir-looking vs. TIR oblique case. The bulk of the feature points was extracted from the ground part, not from the building. These feature points appeared where the brightness value of the pixel changes. For example, the points of area A were elicited from where it changes from the bush to the bare ground. Additionally, a pixel that changes from garden stone to bare ground was drawn as a point. The features gained from the roof of the warehouse located southeast of area A have quite different characteristics. The warehouse was built with prefabricated panels, particularly the roof panel made up of four groove pre-coated steel sheets. Feature points were expressed from the groove of the roof panel, which were perceived as straight stripes in the image processing through PC. In area B, the central part of Figure 9a, the three points emerging from the white straight line represent the road’s border. The white straight line on the road is the point where the pixel value varies greatly.

Another attribute of area B is the absence of buildings. Area B’s region contrasts with the pixels between the trees and the bare ground, so it was predicted that many feature points would be picked. However, as a result of the matching experiment, feature points were not derived. We analyzed the cause of this result because the building’s shadow was included depending on the change in geometry at image acquisition. Pixel values in areas where shadows appear are generally darkened close to black. In other words, matching is complex because notable pixels are not distinguished. Shadows are an inevitable element when obtaining oblique images. It comes into view in sundry directions and forms within the saved image, closely related to the angle of the sensor, the position of the sun, and the time of image acquisition. The improvement of the matching potential would be achieved when setting the UAV’s flight path in detail, such as adjusting the acquisition angle by pre-computing the direction in which the shadow appears.

Figure 9b shows the feature points for the building type, visible oblique vs. TIR nadir-looking case. The case of Figure 9b shows the opposite geometry to Figure 9a. In area A, the mechanism in which each feature point appeared from the ground and roof panel’s groove is similar to Figure 9a. These flows reconfirm that bare ground should exist that is hardy against geometric changes for extracting feature points. Additionally, pixels with differences in brightness compared to surrounding pixels were elicited as feature points, whereas the location of points in area C appeared somewhat differently. Feature points were presented from the roof’s corners and border of the window and fence on the building’s facade. The properties of these points do not occur in Figure 9a and are dependent on the style and configuration of the buildings and roofs. Therefore, there was a setback in the generalization of typical characteristics of points between images with different geometries and wavelengths.

Finally, we formulated an all-embracing solution that can enhance the matching accuracy of images with different geometries and wavelengths containing buildings through the feature points in Figure 9. First, matching is beneficial when the bare ground is placed between buildings. Matching is better if there is an object such as a bush or garden stone with apparent pixel distinction on the ground. Second, the presence of linear objects that are effortless to extract through the maximum value of PC raises the probability of success in matching. Therefore, when acquiring a UAV image, the research area should be prudently set up to incorporate many-sided road markings in an urban area. Third, shadows that inevitably occur when obtaining oblique images should be minimized. For this, the direction of the shadow according to the sun’s location and the building’s height must be computed before image acquisition. Additionally, it is crucial to set the sensor’s angle and the UAV’s flight path minutely. The three abovementioned cores can provide insight into image matching acquired by UAVs with different geometries and wavelengths containing buildings.

Figure 10 is a feature point of a non-building type consisting of fields analogous to a rural environment. In such a situation, points in which differences in pixels appear are scarce, making it more challenging to extract feature points. Additionally, our research area has mountains northwest of the village. Therefore, although there are no objects, such as a building, remarkably affected by geometry, the form was displayed differently due to the altitude change’s repercussion. We divided the feature points into two groups, areas A and B, depending on the placement of the points, to elucidate the characteristics of the features.

Figure 10a shows the feature points for the non-building type of visible nadir-looking vs. TIR oblique case. The feature points’ characteristics were identified by dividing them into two areas, area A on the left with a large field and area B on the right with the house. Area A is tangled with unharvested cabbage, agricultural vinyl, grass, twigs, and dry bushes in roomy farmland. Additionally, this area has furrows, so the bumpy texture is expressed in the image. Feature points were derived from places with a significant difference in pixel values, such as between cabbage and furrows or between dry bushes and furrows. The area was expected to have many points due to its wide furrows. However, the results of extracting the bumpy part of the furrows from the visible and TIR images through the maximum moment of PC were unconnected. The TIR image showed an almost crooked form of striations, and the visible image had no salient features. Therefore, although there were many furrows, the number of points calculated was barely enough to count. Meanwhile, area B, on the right side of the image, has fences and fields adjacent to the house and a waterway to the north. Feature points were elicited from the straight part of the fence, the waterway boundary, and from pixels that change from field to bare ground.

Figure 10b presents the feature points for the non-building type of visible oblique vs. TIR nadir-looking case. This case has the opposite geometry of Figure 10a. In area A, where furrows exist, points were extracted where the pixel values change in the same way as the mechanism mentioned earlier. Area B’s points were expressed from the straight part of the fence and the pixels that change from grass to bare ground, similar to Figure 10a.

Finally, we achieved a profitable solution that can enhance the matching accuracy of images with different geometries and wavelengths under non-building conditions through the feature points in Figure 10. First, the presence of areas such as uneven furrows helps the matching process. We already conducted and identified that images obtained from different wavelengths are somewhat challenging to recognize as the same features, despite having bumpy areas. Therefore, matching with distinct wavelengths and geometries may not be possible if the image only holds the flush area. Second, a flat road made of cement is more challenging to elicit feature points. We previously proved that no feature points appeared from the cement-paved road crossing in the A and B regions, as shown in Figure 10. This property correlates with the notion of bumpy and flush areas mentioned earlier. Third, it is efficient to include well-defined terrain features found relatively easily in non-urban areas when acquiring images by a UAV. In other words, fences, banks, and waterways around the farm were processed as outstanding features. Utilizing these objects helps to improve matching accuracy in rural conditions with fewer formalized shapes, such as crosswalks, traffic lanes, and intersections, compared to urban areas. These three characteristics can perceive image matching obtained by UAVs with different geometries and wavelengths in rural areas.

### 4.3. Accuracy Evaluation

In this study, we aimed to determine the reliability of matching results by performing an accuracy evaluation. As mentioned previously, the experts’ manual selection was performed and assumed to be the ground truth. Then, the RMSE was calculated by applying it to each matching result. Table 6 shows the RMSE of the matching results in pixels. The accuracy of SIFT, SURF, SAR–SIFT, and ASIFT without matching is meaningless, but each RMSE is presented for quantitative comparison with the proposed method. Furthermore, we finally classified the performance of matching results as ‘matched’ and ‘not matched’, according to the experimental results.

Through the accuracy evaluation, the proposed matching method demonstrated superior performance in all types and cases. As shown in Table 6, SIFT, SURF, SAR–SIFT, and ASIFT showed an accuracy of approximately 100 to 400 pixels, but our method indicated about 20 pixels. We have applied projective transformation together with affine transformation to evaluate the accuracy of the proposed method. As a result, the RMSE based on projective transformation averaged about 19 pixels, which was similar to the result obtained in affine transformation. These values may be determined at a lower accuracy than the results among visible nadir images, which are typical matching types. However, it has meaningful value because it overcomes limitations that have not been solved by the popular matching method.

Eventually, we achieved a systematic approach in solving a complex problem, which combined with different geometries and wavelengths and even demonstrated the properties of extracted feature points. In this sense, the proposed method could be a good candidate for a reliable solution.

## 5. Conclusions

The main contribution of this study is matching visible and TIR images with different geometries. Various image matching methods have been offered, but ultimate cases, such as visible nadir-looking vs. TIR oblique and visible oblique vs. TIR nadir-looking, had not yet been realized. To accomplish this, we proposed a new matching method called phase congruency with histogram–IMAS (PCH–IMAS) and compared it with the frequently used image matching methods SIFT, SURF, SAR–SIFT, and ASIFT. The method proposed in this study showed peerless results in both building and non-building types of all cases. Our method is an unrivaled solution that empowers robust feature point extraction in extreme matching situations with different geometries and wavelengths obtained by UAVs. Therefore, our proposed methods that extract maximum moments of images through PC and adjust histograms using histogram matching to match images of different wavelengths and applying IMAS to match distinct geometries is the best combination and reasonable solution.

However, we were not satisfied with the success of matching and discreetly checking the location characteristics of the extracted feature points. We presented three generalized guidelines for building and non-building types to increase the possibility of matching. These standards were understood as logical keys for matching images with different geometries acquired from visible and TIR sensors. The matching accuracy of the proposed method is about 20 pixels, which is highly valuable compared to other methods that are not matched. Finally, the matching of unusually complex cases was successful and has immense significance.

In present-day cities, information and events to pinpoint and monitor are the peaks of day and night. TIR images can obtain information that cannot be perceived with visible images. They are needed universally in many places where information cannot be obtained through human eyes. Thus, an integrated analysis with visible images is essential. The process of utilizing TIR images obtained by UAVs is likely to accelerate soon. This research is state of the art in its approach to image matching, combined with the use of different wavelengths and geometries. In the near future, it will serve as a trustworthy solution and positive strategy for the uptake of TIR imagery.

## Figures and Tables

**Table 1:** Specification of the visible image sensor.

Setup Overview	Detail
Equipment	UAV (MAVIC 2 Enterprise Dual)
Overlap	70%
Average flight height	70 m
Ground Sample Distance (GSD)	2.17 cm (Nadir-looking) 2.48 cm (Oblique)
Oblique angle	30°

**Table 2:** Specification of the TIR image sensor.

Setup Overview	Detail
Equipment	UAV (Inspire 1 + Zenmuse XT camera)
Overlap	70%
Average flight height	70 m
GSD	8.5 cm (Nadir-looking) 10.2 cm (Oblique)
Oblique angle	30°

**Table 3:** Category of experimental images.

No.	Type	Case
1	Building	Visible nadir-looking vs. TIR oblique
2		Visible oblique vs. TIR nadir-looking
3	Non-building	Visible nadir-looking vs. TIR oblique
4		Visible oblique vs. TIR nadir-looking

**Table 4:** The software environments of matching methods.

Method	Operating System (64 bit)	Language	Implementation
SIFT	Windows 10 Pro	MATLAB R2019b	OpenCV
SURF	Windows 10 Pro	MATLAB R2019b	OpenCV
SAR–SIFT	Windows 10 Pro	MATLAB R2019b	Downloaded from GitHub and modified
ASIFT	Linux Ubuntu	C/C++	Downloaded from the path contained in the paper
Proposed	Windows 10 Pro	MATLAB R2019b	Generates code for all sections except IMAS
	Linux Ubuntu	C/C++	Downloaded from the path contained in the paper

**Table 5:** The number of inliers according to the results of matching methods.

No.	Type	Case	Method	Number of Inliers
1	Building	Visible nadir-looking vs. TIR oblique	SIFT	Non-existent
			SURF	Non-existent
			SAR–SIFT	Non-existent
			ASIFT	Non-existent
			Proposed	24
2		Visible oblique vs. TIR nadir-looking	SIFT	Non-existent
			SURF	Non-existent
			SAR–SIFT	Non-existent
			ASIFT	Non-existent
			Proposed	32
3	Non-building	Visible nadir-looking vs. TIR oblique	SIFT	Non-existent
			SURF	Non-existent
			SAR–SIFT	Non-existent
			ASIFT	Non-existent
			Proposed	17
4		Visible oblique vs. TIR nadir-looking	SIFT	Non-existent
			SURF	Non-existent
			SAR–SIFT	Non-existent
			ASIFT	Non-existent
			Proposed	22

**Table 6:** RMSEs of pixel distance based on matching results.

No.	Type	Case	Method	RMSE (Unit: Pixel)	Performance
1	Building	Visible nadir-looking vs. TIR oblique	SIFT	360.46	Not matched
			SURF	402.69	Not matched
			SAR–SIFT	197.41	Not matched
			ASIFT	none	Not matched
			Proposed	22.56	Matched
2		Visible oblique vs. TIR nadir-looking	SIFT	264.80	Not matched
			SURF	240.57	Not matched
			SAR–SIFT	303.72	Not matched
			ASIFT	334.56	Not matched
			Proposed	21.73	Matched
3	Non-building	Visible nadir-looking vs. TIR oblique	SIFT	131.81	Not matched
			SURF	325.98	Not matched
			SAR–SIFT	219.61	Not matched
			ASIFT	none	Not matched
			Proposed	26.25	Matched
4		Visible oblique vs. TIR nadir-looking	SIFT	323.37	Not matched
			SURF	288.68	Not matched
			SAR–SIFT	121.70	Not matched
			ASIFT	none	Not matched
			Proposed	29.01	Matched

## Data Availability

Not applicable.
